# Inhibition of Wnt-β-Catenin Signaling by ICRT14 Drug Depends of Post-Transcriptional Regulation by HOTAIR in Human Cervical Cancer HeLa Cells

**DOI:** 10.3389/fonc.2021.729228

**Published:** 2021-10-28

**Authors:** Samuel Trujano-Camacho, David Cantú-de León, Izamary Delgado-Waldo, Jossimar Coronel-Hernández, Oliver Millan-Catalan, Daniel Hernández-Sotelo, César López-Camarillo, Carlos Pérez-Plasencia, Alma D. Campos-Parra

**Affiliations:** ^1^ Postgraduate in Experimental Biology, DCBS, Autonomous Metropolitan University-Iztapalapa, Iztapalapa, Mexico; ^2^ Laboratorio de Genómica, Instituto Nacional de Cancerología (INCan), Ciudad de México, Mexico; ^3^ Unidad de Investigaciones Biomédicas en Cancerología, Instituto Nacional de Cancerología (INCan), Ciudad de México, Mexico; ^4^ Laboratorio de Epigenética del Cáncer, Facultad de Ciencias Químico Biológicas, Universidad Autónoma de Guerrero, Chilpancingo de los Bravo, Mexico; ^5^ Posgrado en Ciencias Genómicas, Universidad Autónoma de la Ciudad de México, Ciudad de México, Mexico; ^6^ Unidad de Biomedicina, Facultad de Estudios Superiores Iztacala, Universidad Nacional Autónoma de México (UNAM), Tlalnepantla, Mexico

**Keywords:** cervical cancer, Wnt-β-catenin, HOTAIR, ICRT14 drug, post-transcriptional regulation

## Abstract

**Background:**

In Cervical cancer (CC), in addition to HPV infection, the most relevant alteration during CC initiation and progression is the aberrant activation of Wnt/β-catenin pathway. Several inhibitory drugs of this pathway are undergoing preclinical and clinical studies. Long non-coding RNAs (lncRNAs) are associated with resistance to treatments. In this regard, understanding the efficiency of drugs that block the Wnt/β-catenin pathway in CC is of relevance to eventually propose successful target therapies in patients with this disease.

**Methods:**

We analyzed the levels of expression of 249 components of the Wnt/β-catenin pathway in a group of 109 CC patients. Three drugs that blocking specific elements of Wnt/β-catenin pathway (C59, NSC668036 and ICRT14) by TOP FLASH assays and qRT-PCR were tested *in vitro* in CC cells.

**Results:**

137 genes of the Wnt/β-catenin pathway were up-regulated and 112 down-regulated in CC patient’s samples, demonstrating that this pathway is dysregulated. C59 was an efficient drug to inhibit Wnt/β-catenin pathway in CC cells. NSC668036, was not able to inhibit the transcriptional activity of the Wnt/β-catenin pathway. Strikingly, ICRT14 was neither able to inhibit this pathway in HeLa cells, due to HOTAIR interaction with β-catenin, maintaining the Wnt/β-catenin pathway activated.

**Conclusions:**

These results demonstrate a mechanism by which HOTAIR evades the effect of ICRT14, a Wnt/β-catenin pathway inhibitory drug, in HeLa cell line. The emergence of these mechanisms reveals new scenarios in the design of target therapies used in cancer.

## Introduction

Cervical cancer (CC) constitutes a major health concern worldwide since it is the fourth most common cancer in women (1). Epidemiological and molecular studies have shown that high-risk Human Papilloma Virus (HR-HPV) is a causal agent but not sufficient (2). Hence, the malignant progression of HR-HPV infected cells is a casual event that requires the emergence of other genetic, epigenetic, phenotypic and micro-environmental alterations (3–5). Among the most relevant alterations during CC initiation and progression is the aberrant activation of Wnt/β-catenin pathway, which is essential in cervical oncogenesis (6, 7). Wnt/β-catenin pathway has a critical role in development, differentiation, and tissue homeostasis. However, in several types of cancer this pathway is dysregulated promoting poor patient prognosis, so it is attractive to be pharmacologically blocked (8). Only a few drugs have made it into Phase I clinical trials, such as Ipafricept and vantictumab (WNT antibodies), LGK974 and ETC-159 (PORN inhibitors), PRI-724 and CWP232291 (β-catenin inhibitors); however, none has been approved yet (9). Other drugs are still in pre-clinical studies, for example; C59, acts at the extracellular level inhibiting the function of PORCN, which is a membrane-bound O-acyltransferase required for palmitoylation, secretion and activity of WNTs ligands (10). NSC668036, is an organic molecule that acts at the cytoplasmic level binding to DVL protein, that inhibits the Wnt3A induced signaling (11). Another drug is ICRT14, which acts at the nuclear level inhibiting direct interactions between β-catenin and TCF4, antagonizing the transcriptional function of nuclear β-catenin and consequently shutting down the signaling pathway (12). Due to the existence of an arsenal of drugs blocking Wnt/β-catenin pathway, some characteristics that determine their efficacy are becoming apparent (13).

Recent studies have revealed that the dysregulation of multiple pathways by long-non coding RNAs (lncRNAs) results in drug resistance (14, 15). HOX transcript antisense intergenic RNA (HOTAIR) is the best example, as its overexpression induced cellular resistance to cisplatin through Wnt/β-catenin pathway activation in ovarian cancer (16). Likewise, HOTAIR upregulation was associated with drug resistance by Wnt/β-catenin pathway activation in lung cancer (17), and colorectal cancer (18).

In the present study, we validated the dysregulation of Wnt/β-catenin pathway signaling in CC patients. Next, since there are several drugs to inhibit this pathway, we performed *in vitro* assays to determine the efficacy of C59, ICRT14 and NSC668036 in order to inhibit Wnt/β-catenin signaling pathway in CC cell lines (HeLa, SiHa and CaSki). C59 was an efficient drug, NSC668036 showed no inhibitory effect while, ICRT14 turned out to have an inhibitory effect in SiHa and CaSki cell lines but did not inhibit the Wnt/β-catenin pathway in the HeLa cell line. HOTAIR overexpression was verified in HeLa cells, and its potential interaction with β-catenin, was associated with Wnt/β-catenin activation, decreasing ICRT14 drug efficiency. These data revealed a new resistance mechanism, hence, some target therapies are not convenient against cancer due to the lncRNAs-mediated regulation in order to promote drug-resistance. The description of these mechanisms provides new insights into further therapeutic opportunities in CC.

## Materials and Methods

### Cervical Samples

We included 109 cervical cancer patients from 2010 to 2013 through Instituto Nacional de Cancerología of Mexico City (INCan). This study was approved by INCan’s Review Board and Ethics Committee (015/012/IBI-CEI/961/15). All patients of this study agreed and signed the consent form. In order to identify mRNAs deregulated and involved in the Wnt/β-catenin pathway, gene expression microarray assay was performed for which 89 samples were used and 20 were selected to perform validation by qRT-PCR. After surgical excision, tumor biopsies were segmented into two pieces, one for pathological confirmation and another for nucleic acid separation. Twenty non-pathological cervical tissues were obtained from patients who had undergone a hysterectomy due to uterine myomas.

### RNA Purification and Microarray Hybridization

RNA was extracted from 89 cervical cancer samples and 6 cervical non-tumor tissues to perform the microarray assay and it´s quality was measured using the 18S:28S ratio. Microarray was performed as previously reported and the raw data are publicly available at the GEO database (Gene Expression Omnibus, http://www.ncbi.nlm.nih.gov/geo/) with the accession number GSE56303.

### RT−qPCR

RNA from 20 samples tumor tissues and CC cell lines, was extracted with Trizol reagent (Ambion) according to the manufacture’s protocol. The total cDNA was generated by reverse transcription from 1µg of total RNA using the kit High-Capacity cDNA Reverse Transcription (Applied Biosystems) with a final volume of 20 µl. To amplify, c-Jun, c-Myc, MMP7, Cox2, CyD1, MMP10, CTNNB, CSNK1, FZD5, DVL, LRP5, NKD2 Klotho (KL), Cerberus (CER1), NKD1, Wnt11 and HOTAIR, a Luminaris Color HiGreen qPCR Master Mix was used along specific primers and specific amplification conditions for each gene (**Supplementary File 1**). Reactions were performed in Step One System. Relative expression levels were calculated using the ΔΔCt method (Applied Biosystems). β-actin mRNA was used as a reference gene for normalization. At this section it is important to emphasize that the idea was to evaluate the expression of each of these genes in each tumor and normal sample. However, the amount of RNA obtained from each of the samples (tumor) was insufficient to do so. Therefore, of the 20 tumor tissue samples, only 19 were evaluated for c-Jun expression, 14 for NKD expression and 13 for DVL expression.

### Cell Culture, Transfection, and Reagents Drugs

All cell lines were obtained from ATCC. Human CC cell lines, HeLa (ATCC CRM-CCL-2) and SiHa (ATCC HTB-35) were cultured in DMEMF12 (Gibco) medium while CaSki cell line (ATCC CRL-1550) was cultured in RPMI (Gibco) medium, both mediums were supplemented with 10% (v/v). All cells were maintained at 37°C in a humidified atmosphere with 5% CO2. The drugs C59 (Bio-vision 2063-5) and ICRT14 (Toronto Research Chemical Canada I163900) were purchased from Sigma Aldrich and were solubilized in dimethyl sulfoxide (DMSO; Sigma-Aldrich, St. Louis, MO, USA). NSC668036 (TOCRIS BIOSCENSE 5813) was purchased from TOCRIS and was dissolved in water. All reagents were stored at -20°C. The IC50 (concentration resulting in a 50% inhibition of cell growth) for each drug is provided by the supplier.

### Viability MTT Assay

In order to verify the half maximal inhibitory concentration (IC50) provided by the supplier for C59, NSC668036 and ICRT14 in HeLa, SiHa and CaSki cell lines, an MTT assay was employed to determine cell viability. Briefly, 4x 10^3^ cells were seeded in a 96-well plate. After 24 hrs of incubation, the cells were exposed to different concentrations close to those given by the supplier of C59, NSC668036, ICRT14 and DMSO as a control in fresh medium for 24 hrs. Cells were washed with PBS and were exposed to MTT (300 μL/well, 1 mg/mL; Sigma) for 3 hrs at 37°C. Then, cells were washed and incubated with 100 µL of DMSO for 10-15 min. Finally, the optical density (OD) was recorded at 540 nm in an Epoch Microplate Spectophotometer (Bioteck).

### TOP/FOP Flash Assay

To determine the activity of Wnt/β-catenin pathway, TOP/FOP flash assay (TCF Reporter Plasmid Kit Merck Millipore) was performed following the manufacturer’s instructions. Briefly, 4x10^5^ cells were seeded in a 6-well plate and co-transfected with 2.5 µg of TOP and FOP plasmids. After 24 hrs, cells were incubated with the IC50 of each inhibitor (C59, NSC668036, ICRT14) or 30 µM DsiHOTAIR (IDT; San Diego, CA, USA) and a scramble sequence (scramble silencer negative control Ambion AM4611); using Lipofectamine 2000 transfection agent (Invitrogen). After incubation for 24 hrs, the cells of each group were collected, and then the activity of Wnt/β-catenin signaling pathway was measured by Dual Luciferase Reporter Assay Kit (Promega) in GloMax**^®^** 96 Microplate Luminometer (Promega; Madison, WI, USA).

### Flow Cytometry for Annexin V/Propidium Iodide (PI)

Apoptosis was assessed by staining cells with annexin V-fluorescein isothiocyanate (FITC) and propidium iodide (PI). Briefly, HeLa cells were washed with PBS, and suspended in serum-free, phenol red-free medium. HeLa cells were seeded in 6-well plates at a density of 3x10^5^ cells/well. After 24 hrs, the cells were incubated either with the scramble (30 µM for 48 hrs), Dsi HOTAIR (30 µM for 48 hrs), ICRT14 (IC50 12.9 µM for 24 hrs) or the combination DsiHOTAIR (30 µM for 48 hrs) plus ICRT14 (IC50 12.9 µM incubated for this condition for 24 hrs). Then, the cells were washed with PBS. The level of annexin V binding was determined by using a commercially available annexin V apoptosis detection kit (FITC Annexin V Apoptosis Detetion Kit with PI, BioLegend), according to the manufacturer´s instructions. The cells were subsequently analyzed by a flow cytometer (FACScalibur). Approximately 10,000 events were collected for each sample. The percentage distributions were calculated by Expo32 ADC software (Beckman Coulter, Inc., Miami, FL). Cells were classified as apoptotic (positive annexin V and negative PI), late apoptotic/necrotic (positive annexin V and positive PI) or viable (negative annexin V and PI). Unstained HeLa cells were used as negative fluorescence controls. The same procedures were performed for 30 μg/ml etoposide treated cells. Moreover, we captured a photography for each condition (Microcopy leica 090-135.002).

### RNA Binding Protein Immunoprecipitation (RIP) assay

RNA immunoprecipitation (RIP) was performed using Magna RIP RNA-Binding Protein Immunoprecipitation kit (17-704, EMD Millipore) according to the manufacturer´s instructions. HeLa cells were lysed in complete RIP lysis Buffer, after the lysate was incubated with RIP buffer containing magnetic beads conjugated with 2.5 µg to human Anti- β-catenin (Abcam, ab227499) and negative control normal rabbit IgG (Millipore). Samples were incubated with proteinase K and the inmunoprecipitated RNA was isolated. Finally, HOTAIR was amplified by qRT-PCR as mentioned before.

### Protein Expression Analysis

Protein extracts from cultured cells were achieved by homogenization in RIPA buffer (Santa Cruz Biotechnology), later dissipated by centrifugation at 12,000 rpm for 20 min. For immunodetection, 50 µg total protein from cultured cells were mixed with Laemmli sample buffer, boiled, separated in 12 or 15% SDS-PAGE, and transferred in a PVDF membrane (Amersham-GE Healthcare). Membranes were incubated overnight using a 1:1,000 (v/v) dilution of the anti-caspase 3 (Cell signaling), anti-PARP46D11 (Cell signaling), anti-c-Jun (Cell signaling) and anti-c-Myc (Cell signaling). For detection, 1:2,500 (v/v) dilutions of HRP anti-rabbit or anti-mouse conjugate antibodies (Santa Cruz Biotechnology) were used. Finally, using the Super Signal West Femto chemiluminescent substrate (Thermo Scientific), the membranes were scanned in the C-Digit blot scanner (Li-Cor) and the images were analyzed for densitometry in the associated Image Studio software (LiCor). Membranes were stripped and re-probed for detection of actin (anti-actin, Sc-47778) as a loading control. A representative image from three independent experiments is shown.

### Bioinformatics Analysis

RPISeq software (from website http://pridb.gdcb.iastate.edu/RPISeq/) was used to predict the interaction probability between HOTAIR and β-catenin protein. The interaction probability accepted was ≥ 0.8 in both classifiers of random forest (RF) and support vector machine (SVM).

### Statistical Analysis

In order to obtain a list of significant genes from Wnt signaling pathway aberrantly expressed in tumor tissues *versus* their normal counterparts, we used significance analysis of microarrays (SAM) software. This software assigned a score based on the change of expression relative to the standard deviation of repeated measurements of each Wnt pathway-dysregulated components. Genes with scores higher than the threshold are considered potentially significant, in this way, we contemplated as positively or negatively regulated genes those with a delta score >0.3 and less than − 2.0, respectively (19). All data are expressed as the mean ± S.D. from three independent experiments. Statistical analyses were performed using one-way ANOVA. P < 0.05 (*) or P < 0.01 (**) was considered to indicate statistical significance.

## Results

### Patients

One hundred nine patients were recruited. Of these, 89 patients were used to assess mRNA profile expression through a microarray assay and 20 were selected for RT-qPCR validation of the data generated by the microarray. The mean age of the patients was 48 years (range, 29-70 years). All patients were diagnosed with CC and the most common histologic subtype was squamous cell carcinoma (90.8%). According to the clinical stage classification (FIGO), patients’ specimens were categorized as follows; 60.5% stage IIB, 24.7% stage IIIB, 12% stage IB2 and 0.91% stage IIA and IIIA.

### Wnt Signaling Pathway Is Deregulated in Cervical Cancer Patients

**Figure 1:**
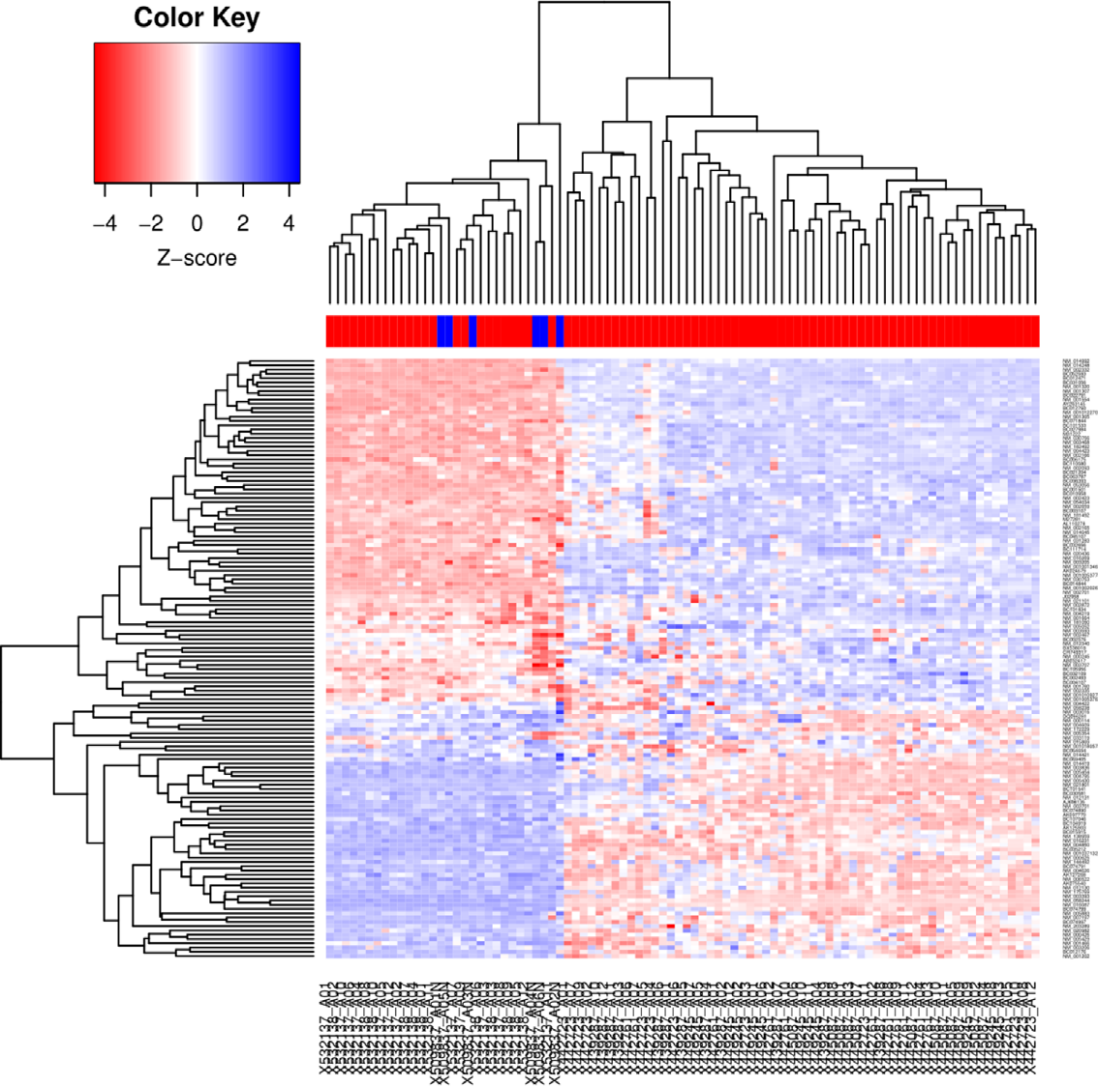
Hierarchical cluster generated from 89 LACC and 6 non-tumor tissue samples. Cluster analysis of the microarray data. The microarray data were analyzed by the Genesis program. The cluster shows 249 genes (137 up-regulated and 112 down-regulated). Each row represents a gene, whereas each column corresponds to a tissue sample, the color line above the tissue samples indicates the sample type: normal samples (blue) and tumor samples (red). The relative abundance of each gene in the tissue correlates with the color intensity (red, induced; blue, repressed; white, no change). In the dendrogram, all six normal cervical samples clustered together, indicating their similarity based on the expression profile.

As a first approach, in order to identify the differential expression genes involved in Wnt/β-catenin signaling pathway of CC specimens, microarray data was analyzed with SAM algorithm (https://statweb.stanford.edu/~tibs/SAM/), which detects genes with important expression changes using Delta Score (Score (d) ≥ 1.5 and ≤ 1.5 and a false discovery rate (FDR) < 10%). Thus, we analyzed 249 genes and isoforms involved in Wnt signaling pathway which expression was significantly altered in CC specimens compared to normal cervical tissues (137 were up-regulated and 112 down-regulated) (**Supplementary File 2**). In **Figure 1** is shown a hierarchical clustering in which Pearson correlation distance and complete linkage clustering were used to display differences and similarities based on the expression profiles obtained from Genesis 2.1 software (20).

**Figure 2:**
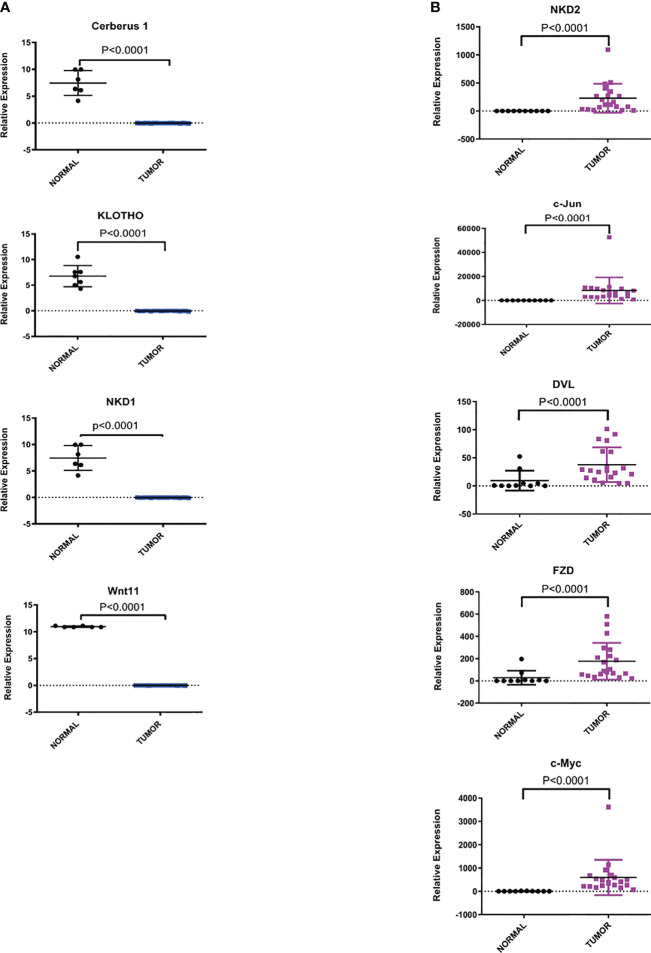
Relative expression of dysregulated genes of Wnt/β-catenin pathway in CC samples *versus* normal cervix tissues. The expression level of each gene by RT-qPCR was determined as described in the methods section. Statistical analysis to compare the mRNA expression levels between normal and tumor tissues was performed using an unpaired two-tailed t-test. **(A)** Representative downregulated genes of the Wnt/β-catenin pathway. **(B)** Representative upregulated genes of the Wnt/β-catenin pathway.

To confirm the microarray data, we perform RT-qPCR to validate the expression of key upregulated genes such as: NKD2, c-Jun, DVL, FZD5 and c-Myc as well as key downregulated genes such as: Cerberus (CER1), Klotho (KL), NKD1 and Wnt11. The expression was evaluated in an independent cohort of 20 cervical cancer specimens and 10 normal cervical tissues. As shown in **Figure 2**, the expression levels of each gene obtained by RT-PCR correlated and were consistent with the microarray data analysis.

### C59, NSC668036 and ICRT14 Reducing the Cell Viability in a Dose-Dependent Manner

**Figure 3:**
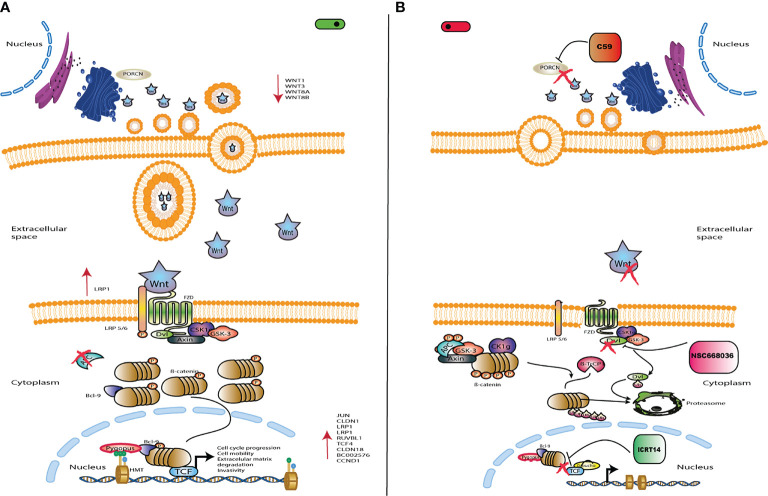
Schematic representation of Wnt/β-catenin signaling. **(A)** Wnt/*β-*catenin signaling is activated by Wnt ligands, which bind to the FZ/LRP5/6 receptor in order to inhibit the destruction complex. Thus, leading to accumulation of cytoplasmic β-catenin and its eventual translocation into the nucleus. Then, β-catenin binds to TCF/LEF sites to activate transcription of target genes such as CCND1 and MYC, which are involved in cell proliferation and survival. **(B)** The drug C59 acts at the extracellular level inhibiting the Wnt ligands to block the pathway. NSC668036 acts at the cytoplasmic level binding to DVL protein, which inhibits the Wnt3A induced Wnt/β-catenin signaling. ICRT14 acts at the nuclear level inhibiting direct interactions between b-catenin and TCF4, blocking the transcriptional function of nuclear β-catenin. Left panel shows the Wnt/β-catenin signalling pathway turned on; while right panel turned off.

**Figure 4:**
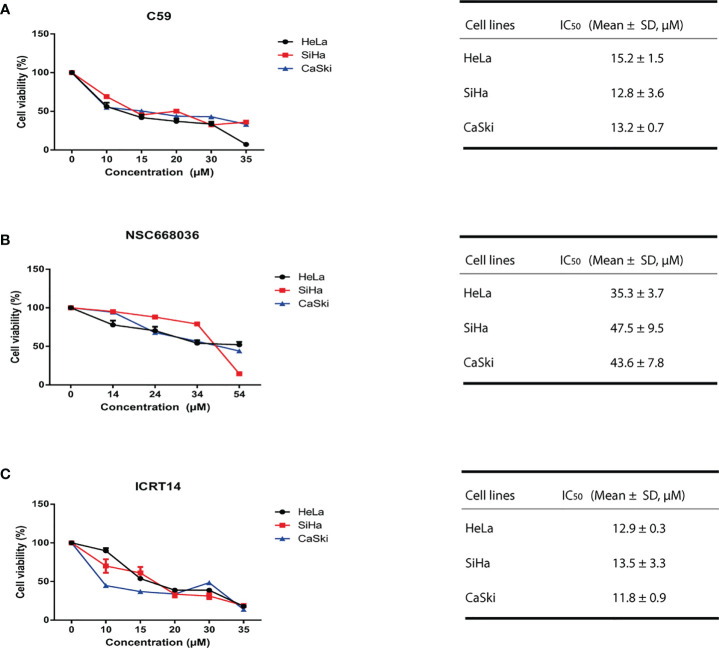
C59, NSC668036 and ICRT14 decrease proliferation of CC cells. HeLa, SiHa and CaSki cells were treated with different doses of **(A)** C59, **(B)** NSC668036 and **(C)** ICRT14 for 24 hrs. Cell viability was analyzed using MTT. IC50 was determined by non-linear regression.

One of our main goals was to probe the efficacy of three drugs (C59, NSC668036 and ICRT14) to inhibit the Wnt/β-catenin pathway at three different levels (extracellular, cytoplasm and nucleus) into the CC cells (**Figure 3**). For that purpose, first we verify the IC50 provided by the supplier of each drug. Thus, we tested a range of concentrations for each drug in HeLa (epithelial adenocarcinoma CC cell line infected with HPV18), SiHa and CaSki (both are squamous cancer cell line infected with HPV16 cells). As expected, the results shown in **Figure 4** indicate that these drugs reduced cell survival in a dose-dependent manner. According to the findings, IC50 for C59 and ICRT14 were similar in HeLa, SiHa and CaSki cell lines (**Figures 4A, C**), whereas IC50 for NSC668036 was higher. Therefore, C59, ICRT14 and NSC668036 were selected for subsequent experiments.

### C59 and ICRT14 Inhibit the Transcriptional Activity of Wnt/β-Catenin Pathway in CC Cell Lines

**Figure 5:**
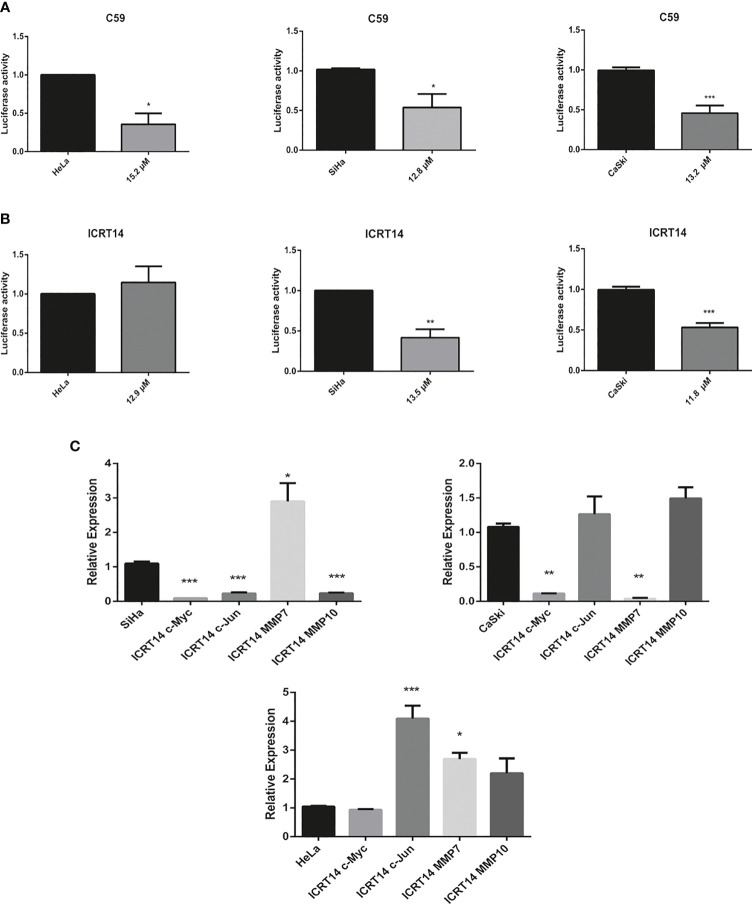
Effect of C59 and ICRT14 drugs on Wnt/β-catenin pathway in CC cell lines. HeLa, SiHa and CaSki cells were co-transfected with 2.5 μg FOPFlash-Luc (mutant reported vector) and TOPFlash (Wnt/β-catenin reporter vector). After 24 hrs, they were incubated with IC50 of **(A)** C59 and **(B)** ICRT14. After 24 hrs, the luciferase activity and the expression of the main targets of wnt pathway was measured in order to determine the Wnt/β-catenin pathway activation by luciferase assay **(A, B)** and **(C)** RT-qPCR, respectively. The bars represent the mean ± standard deviation from at least three independent experiments. *p < 0.05; **p < 0.01 and ***p < 0.001.

In order to determine if C59, NSC668036 and ICRT14 inhibit the Wnt/β-catenin pathway in CC cell lines, we conducted a TOP-flash assay, the standard assay for assessing Wnt/β-catenin pathway activity. The transcriptional activity of Wnt/β-catenin pathway was determined in HeLa, SiHa and CaSki using the IC50 of C59, NSC668036 and ICRT14. As we expected, the results demonstrated that C59, was able to significantly inhibit the transcriptional activity of Wnt/β-catenin in HeLa, SiHa and CaSki cell lines (**Figure 5A**). Moreover, the expression of c-Myc and c-Jun, two main targets of the Wnt/β-catenin pathway, was downregulated when we used C59 in siHa and CaSKi cell lines. In HeLa treated with C59, c-Myc expression protein decreased but not c-Jun expression protein, it is presumed that alternative pathways may be activating its expression (**Supplementary File 3A**). Conversely, NSC668036, was not able to inhibit the transcriptional activity of the Wnt/β-catenin pathway in HeLa, SiHa and CaSki cell lines (**Supplementary File 4**). Regarding ICRT14, was able to inhibit the transcriptional activity of Wnt/β-catenin pathway in SiHa and CaSki cell lines but not in HeLa cell line (**Figure 5B**). Furthermore, to corroborate whether ICRT14 was unable to inhibit the Wnt/β-catenin pathway in HeLa cell line, we analyzed the expression of main targets of the Wnt/β-catenin pathway such as c-Myc, c-Jun, MMP7 and MMP10 in HeLa, SiHa and CaSki treated with ICRT14. The results showed that the expression of c-Myc, c-Jun and MMP10 was downregulated in SiHa cells treated with ICRT14. In CaSki cells treated with ICRT14 was downregulated c-Myc and MMP7. Nevertheless, in HeLa cells treated with ICRT14, c-Myc expression was maintained but c-Jun, MMP7 and MMP10 expression was upregulated (**Figure 5C**). Moreover, the expression protein of c-Myc and c-Jun also was overexpressed when HeLa cells were treated with ICRT14 (**Supplementary File 5A**). These data suggested that the Wnt/β-catenin pathway continues to be active, despite the use of ICRT14. This ICRT14 does not inhibit Wnt/β-catenin pathway in HeLa cells.

### HOTAIR Maintains Active Wnt/β-Catenin Pathway in HeLa Cells Despite Treatment With ICRT14 Drug, by Interaction With β-Catenin

**Figure 6:**
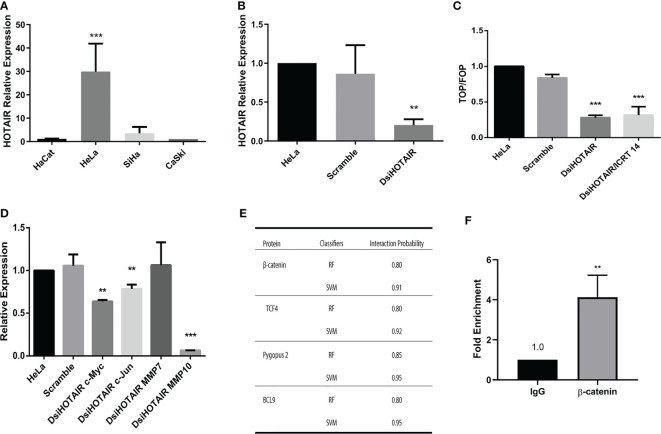
HOTAIR maintains Wnt/β-catenin pathway active in HeLa cells by avoiding the effect of ICRT14. **(A)** Relative expression of HOTAIR was determined by RT-qPCR on CC cells. **(B)** HeLa cells were transiently transfected with 30 µM of DsiHOTAIR. After 48 hrs of post-transfection, the expression of HOTAIR was measured. **(C)** HeLa cells were transiently transfected with 30 µM of DsiHOTAIR alone or in combination with 12.9 µM of ICTR14 and the activity of Wnt/β-catenin pathway was detected by TOPFlash assay at 48 hrs. **(D)** The relative expression of c-Myc, c-Jun, MMP7 and MMP10 was determined by RT-qPCR from HOTAIR knockdown HeLa cells. **(E)** Interaction probability between HOTAIR and β-catenin detected by RPIseq tool. RIPseq uses the classifiers of random forest (RF) and support vector machine (SVM) for calculation. **(F)** Relative RIP assays using qPCR to detect binding between β-catenin and HOTAIR in HeLa cell line.The bars represent the mean ± standard deviation from at least three independent experiments. *p < 0.05; **p < 0.01 and ***p < 0.001.

Several reports have described that upregulation of HOTAIR stimulates the Wnt/β-catenin pathway in several types of cancer including lung (21), pancreatic (22), ovarian (23) and cervical cancer (17); mainly in HeLa cells (24). Therefore, we explored if this mechanism was responsible of the ICRT14 drug inefficiency to inhibit Wnt/β-catenin pathway in the HeLa cell line. First, we aimed to quantify HOTAIR expression by RT-qPCR in CC cell lines and CC biopsies samples. As expected, we found that HOTAIR was significantly upregulated in CC cells lines and CC biopsies samples compared to the non-tumor cell line HaCat and normal tissues samples patients, respectively (**Figure 6** and **Supplementary File 3B**). Moreover, HOTAIR expression was higher in HeLa cell line *versus* SiHa and CaSki cells (**Figure 6A**). Nonetheless, ICRT14 treatment increased HOTAIR expression in HeLa cells (**Supplementary File 3C**). To verify this data, we used a DsiRNA to perform a HOTAIR knockdown in HeLa cell line (**Figure 6B**) and evaluated the effect of ICRT14. Indeed, HOTAIR´s downregulation led to the inhibition of Wnt/β-catenin pathway, also when the ICRT14 drug was added (**Figure 6C**). Besides, three main targets of the Wnt/β-catenin pathway such as; c-Myc, c-Jun and MMP10 expression was downregulated when we used a DsiRNA to perform a HOTAIR knockdown in HeLa cell (**Figure 6D**). Moreover, the expression protein of c-Myc and c-Jun was not downregulated when we used ICRT14 in HeLa cells versus SiHa and CaSki cells lines (**Supplementary Files 5A–C**), but a modest reduction in expression was observed when we used a DsiRNA to perform a HOTAIR knockdown in HeLa cell (**Supplementary File 5A**). These findings indicate that HOTAIR expression in HeLa cells activates or maintains active Wnt/β-catenin and inhibited the blocking effect of ICRT14 on this pathway. Since ICRT14 acts at the nuclear level inhibiting direct interactions between β-catenin and TCF4, blocking the transcriptional function of nuclear β-catenin, it was feasible to hypothesize that HOTAIR was maintaining the interaction between β-catenin and TCF4, blocking the effect of ICRT14. To support this idea, we explored RPIseq tool, that predicts protein-RNA interactions. We found that RPIseq tool predicted interactions between HOTAIR and β-catenin, as well with TCF, PYGO2 and BCL9 (**Figure 6E**). Next, to demonstrate at least one of these interactions, we performed a RIP assay. We found that HOTAIR was highly enriched in β-catenin-RNA precipitates compared to input precipitates (**Figure 6F** and **Supplementary File 6**). These findings suggested a potential interaction between β-catenin and HOTAIR, which could prevent the blocking effect of ICRT14 on Wnt/β-catenin pathway.

### HOTAIR Knockdown Induce Necrosis in HeLa Cell Line Incubated With ICRT14

**Figure 7:**
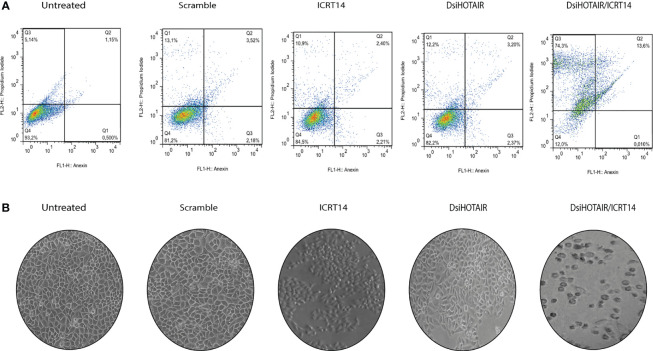
DsiHOTAIR plus ICRT14 induces cell death by necrosis in HeLa cells **(A)** Flow cytometry percentage distributions after annexin V/propidium iodide staining of HeLa incubated with the scramble, DsiHOTAIR (30 µM), ICRT14 (12.9 µM) and the combination DsiHOTAIR/ICRT14. **(B)** Representative images of HeLa cells incubated with the scramble, DsiHOTAIR, ICRT14; and the combination DsiHOTAIR*/*ICRT14.

To identify the mechanisms by which DsiHOTAIR plus ICRT14 decrease Wnt/β-catenin pathway in HeLa cells, we analyzed cell death by flow cytometry assay. As in **Figure 7** is shown, untreated HeLa cells, and HeLa cells transfected with scramble, the rate cell viability was 93% and 89%, respectively. Similarly, when HeLa cells were treated independently with the ICRT14 drug for 24 hrs, and transfected with DsiHOTAIR, the 85% and 83% of cells were viable, respectively (**Figure 7**). These data confirmed our finding obtained with the TOP-flash assay (**Figure 6C**). However, when HeLa cells were incubated with DsiHOTAIR plus ICRT14 drug, only 14% of the cells were underwent apoptosis, and 74% necrosis. (**Figure 7** and **Supplementary File 5D**). Taken together, HeLa cells treated with DsiHOTAIR in combination with ICRT14 drug, induces cell death mainly by necrosis.

## Discussion

Accumulating evidence has reported dysregulation of Wnt/β-catenin signaling in several types of cancer (8). Specifically, in CC, activation of this pathway is a second hit to develop the disease (6, 7), since the transformation of HPV expressing human keratinocytes requires activation of the Wnt/β-catenin pathway (25). In the present study, through a transcriptome exploration on 89 CC samples and 6 non-tumor tissues, we found that Wnt/β-catenin signaling pathway was significantly altered, confirming previous reports (6, 7). We validated the expression of some Wnt/β-catenin pathway involved genes by qRT-PCR in CC specimens. Similar to our findings, some studies have reported the altered expression of DVL (26), FZD5 (27), c-Myc (28), Cox2 (29), c-Jun (30) and Klotho (31). These data support that members of Wnt/β-catenin pathway may be attractive upcoming therapeutic targets (32).

Despite the existence of many drugs to block Wnt/β-catenin (9), it seems that not all of them are effective. This lack of efficacy may be due to drugs acting on different elements of the pathway. It can also be explained by the cellular context in which drugs are being used, even, drug efficacy may be subject to regulation by ncRNAs. C59, NSC668036 and ICRT14 act at extracellular, cytoplasmic and nuclear level, respectively. In the current study, we found that C59 was a highly efficient drug in HeLa, SiHa and CaSki cell lines. Consistent with our results, it has been reported that C59 blocked Wnt/β-catenin pathway, and in consequence, migration and invasion of triple negative breast cancer cells were inhibited (33, 34). In mice, C59 displayed good bioavailability, it did not exhibit toxicity and blocked progression of mammary tumors, suggesting that C59 is a safe and feasible strategy to block Wnt/β-catenin signaling (35). In colorectal cancer, Koo BK and collaborators, demonstrated that C59, attenuated hyperplasias in mouse-small intestinal stem cells (36). Additionally, mice with nasopharyngeal carcinoma treated with C59 did not develop visible tumors. Moreover, it was demonstrated that this agent inhibited the generation of cancer stem cells (CSCs), activity responsible of Wnt/β-catenin signaling (37). It is significant to mention that this is the first study that evaluated the effect of C59 in CC cells. Taken together, is reasonable to consider that small molecule Wnt/β-catenin pathway inhibitors open a new therapeutic window for what should be tested in clinical trials of patients carrying malignant tumors.

Contrary to C59, we found that NSC668036 did not inhibit Wnt pathway in CC cells. Although, NSC668036 has been less explored compared to C59, it was important for us to consider NSC668036 in our study. In concordance to our results, Shin J and collaborators reported that among several agents that block Wnt pathway, NSC668036 was not an efficient drug to block Wnt/β-catenin pathway in HeLa cells (38). Conversely, NSC668036 blocked Wnt/β-catenin signaling in experimental diabetic peripheral neuropathy rats provided with neuroprotection (39). Furthermore, Reshman K. and Sharma S. reported that in rats treated with paclitaxel and NSC668036, their behavioral pain thresholds and nerve functional parameters were significantly improved by inhibition of Wnt/β-catenin signaling (40). Therefore, we speculate that these findings suggest that each inhibitor had differential effects depending on the cellular context.

Interestingly, the agent ICRT14 inhibited Wnt/β -catenin pathway in SiHa and CaSki but not in HeLa cells. The positive effect of this agent is similar to previously reported results. For instance, ICRT14 inhibited c-Myc and cyclin D1 expression in breast cancer cells as well as it decreased migration and invasion (41–43). In colon cancer cells, ICRT14 inhibited Wnt pathway and sensitized cells to radiation treatment (12, 44, 45). In leukemic cell lines, ICRT14 led to significant downregulation of Wnt target genes (46). In the case of lung cancer, ICRT14 is efficient, and even, is used as a positive control to validate new drugs (47). In pancreatic cancer (48), head and neck cancer (49) and Gallbladder carcinoma (50) cells, ICRT14 has also been demonstrated to be an efficient agent.

Surprisingly, we found that ICRT14 had no effect in HeLa cells due to HOTAIR overexpression maintains Wnt/β-catenin pathway activated. Consistent with our results, it was recently reported that HOTAIR is involved in overactivation of Wnt/β-catenin pathway in HeLa cell line (24). Likewise, HOTAIR maintains Wnt/β-catenin activated in esophageal squamous cell carcinoma (51). Additionally, in line with previous reports, we found that HOTAIR is overexpressed in HeLa cells compared to SiHa and CaSki cell lines (52, 53). Thus, HeLa cells have been used as a model to study mechanisms involving HOTAIR in CC (54–57).

We noticed that HOTAIR knockdown in combination with ICRT14 downregulated Wnt/β-catenin pathway in HeLa cells. These results suggest that HOTAIR overexpression conducted to Wnt inhibitors-resistance through Wnt/β-catenin pathway activation. In this regard, it was already known that HOTAIR induces chemoresistance activating Wnt pathway in other types of cancer such as ovarian (16), colorectal (18) and lung cancer (55). In CC cells and in pancreatic ductal adenocarcinoma, HOTAIR knockdown enhanced sensitivity to radiotherapy through Wnt signaling pathway suppression (22, 55). One of the mechanisms of resistance is that HOTAIR promotes β-catenin transportation to the nucleus to maintain the pathway activated (19). In this work, we observed that in HeLa cells line, ICRT14 did not reduced the activation of the Wnt/β-catenin pathway. Since, ICRT14 inhibit the direct interaction between β-catenin and TCF4, blocking the transcriptional function of nuclear β-catenin, we hypothesize that in HeLa cell line, ICRT14 has no effect because HOTAIR is binding to β-catenin to retain it in the nucleus and preserve the pathway active. Although a direct interaction between HOTAIR and β-catenin has not been reported yet, this work is the first to suggests the interaction between them, thus confirming the role of lncRNAs as protein binding scaffolds sustaining the tumoral phenotype and therapy-resistance (20).

We also found that HOTAIR knockdown plus ICRT14 induced cell death mainly by necrosis. Regarding this, it has been reported that apoptosis machinery is defective in numerous cancers (58, 59). Moreover, it is well described that Wnt/β-catenin pathway regulates early and late apoptosis in cancer (60–64). In our study, Wnt/β-catenin pathway was inhibited by ICRT14 in combination of HOTAIR knockdown, consequently, it is reasonable to contemplate that, since the apoptosis machinery was disturbed, alternative pathways of cell death such as necrosis took place. As in ICRT14, it has been reported that anticancer drugs, such as β-lapachone, apoptolidin and honokiol, induce cancer cell death through necrosis (65–67). In this way, necrosis induced by drugs and lncRNAs downregulation, may play an important therapeutic role as the main goal of cancer treatment is, irrevocably, cell death. In conclusion, our results indicate that C59 is a good option as a treatment in CC, although further studies are still required in clinical trials. Moreover, we determined that the effect of ICRT14 in CC depends on the cellular regulation by HOTAIR. These findings indicate that not all target therapies can be efficient and that regulation by lncRNAs should be considered as an alternative treatment for drug resistance mechanisms.

## Conclusions

This is the first study to report the inhibitory effect of C59 on cervical cancer, which was an efficient target therapy for Wnt/β-catenin, *in vitro*. Clinical trials are needed to validate its effectiveness. On the other hand, ICRT14 inhibits direct interactions between β-catenin and TCF4 shutting down the signaling pathways; however according to our results the presence of HOTAIR affected the inhibitory effect of the drug by the potential interaction with β-catenin. These findings demonstrate that the effectiveness of target therapies can be affected by lncRNAs, which have been shown to play an important role in treatment resistance.

## Data Availability Statement

The datasets presented in this study can be found in online repositories. The names of the repository/repositories and accession number(s) can be found in the article/**Supplementary Material**.

## Ethics Statement

The studies involving human participants were reviewed and approved by Instituto Nacional de Cancerología. The patients/participants provided their written informed consent to participate in this study.

## Author Contributions 

AC-P and CP-P conceived and designed the study. ST-C, JC-H, ID-W, and OM-C performed the experiments. AC-P wrote manuscript. DH-S and CL-C contributed to the discussion and analysis of results. DC recollected samples and were responsible for all clinical data of the patients. All authors contributed to the article and approved the submitted version.

## Funding

This research was funded by Instituto Nacional de Cancerología, “Institutional Funds”.

## Conflict of Interest

The authors declare that the research was conducted in the absence of any commercial or financial relationships that could be construed as a potential conflict of interest.

## Publisher’s Note

All claims expressed in this article are solely those of the authors and do not necessarily represent those of their affiliated organizations, or those of the publisher, the editors and the reviewers. Any product that may be evaluated in this article, or claim that may be made by its manufacturer, is not guaranteed or endorsed by the publisher.

## Supplementary Material

Supplementary File 1 Primer sequences.

Supplementary File 2 Genes and isoforms involved in Wnt signaling pathway from CC specimens compared to normal cervical tissues.

Supplementary File 3
**(A)** Relative expression of c-Myc and c-Jun in HeLa, SiHa and CaSKi cells lines treated with C59 drug. **(B)** Relative expression of HOTAIR in normal cervix tissues and CC samples. **(C)** Relative expression of HOTAIR in HeLa, SiHa and CaSki cells treated with C59 and ICRT14 drugs.

Supplementary File 4 Effect of NSC668036 on Wnt/β-catenin pathway in CC cell lines.

Supplementary File 5 Protein levels of c-Myc and c-Jun detected by western blot, in HeLa **(A)**, SiHa **(B)** and CaSki **(C)** treated with ICRT14, C59 and DsiHOTAIR. β-actin was used as loading control. **(D)** Protein levels of PARP1 and Caspase 3 detected by western blot in HeLa cells treated with ICRT14, DsiHOTAIR and DsiHOTAIR plus ICRT14 combination. β-actin was used as loading control.

Supplementary File 6 Western blot to IP β-catenin confirmation of RIP assays. Anti-IgG served as negative control.
