# Supplementary material for: Inhibition of Wnt-β-Catenin Signaling by ICRT14 Drug Depends of Post-Transcriptional Regulation by HOTAIR in Human Cervical Cancer HeLa Cells
Source: Front Oncol. 2021 Oct 28;11:729228. doi: 10.3389/fonc.2021.729228 (PMC8580948; doi:10.3389/fonc.2021.729228)

Supplementary Material

Supplementary File 1. Primer sequences.

Oligonucleotide	Forward	Reverse	Temperature
**Cerberus 1 (CER1)**	CTCCTTTGGGCCTCATCATTTA	GTCTTTCCTAGAGGCAGGAGTA	58 ºC
**Klotho**	AATAATGCCTAGTGGCTTCCC	CTCTGTCGTCTCTCCTGTATCT	57 ºC
**NKD1**	GCCTTGGTGGTGTATGAGAG	CTGGTAGAAGTGGTGGTAATGG	60 ºC
**Wnt11**	AGGACTCGGAACTCGTCTAT	TGTTGCACTGCCTGTCTT	60 ºC
**NKD2**	ACATGGCGGTGAACACATCT	CCTACAGCAGTCACCCCTTG	60 ºC
**c-Jun**	AAG AAC TCG GAC CTC CTC ACCTCG	GCC CTC CTG CTC ATC TGT CAC GTT	63ºC
**DVL**	GAC CGC ATG TGG CTC AAG A	GAG AAG GTG ATC TTG TTG AC	58ºC
**FZD5**	TGC CCA TTC TGA AGG AGT CACACC C	GAG ATG AAG CAC AGC ACC GACCAC	63ºC
**c-Myc**	TTT TGC CCT GCG TGA CCA GATCCC	CTC GTC GTT TCC GCA ACA AGTCCT	64ºC
**MMP7**	ATC ATG ATT GGC TTT GCG CGAGGA	TCC CTA GAC TGC TAC CAT CCGTCC A	63°C
**MMP10**	CAAGGATCTTGCCCAGCAATAC	TTCCCTGTCACCTCCAACCC	64°C
**HOTAIR**	GGTAGAAAAAGCAACCACGAAGC	ACATAAACCTCTGTCTGTGAGTGCC	60°C
**Actin**	ATGACTTAGTTGCGTTACACCCT	TGCTCGCTCCAACCGACTG	60°C

Supplementary File 3. (A) Relative expression of c-Myc and c-Jun in HeLa, SiHa and CaSKi cells lines treated with C59 drug. (B) Relative expression of HOTAIR in normal cervix tissues and CC samples. (C) Relative expression of HOTAIR in HeLa, SiHa and CaSKi cells treated with C59 and ICRT14 drugs.

A)

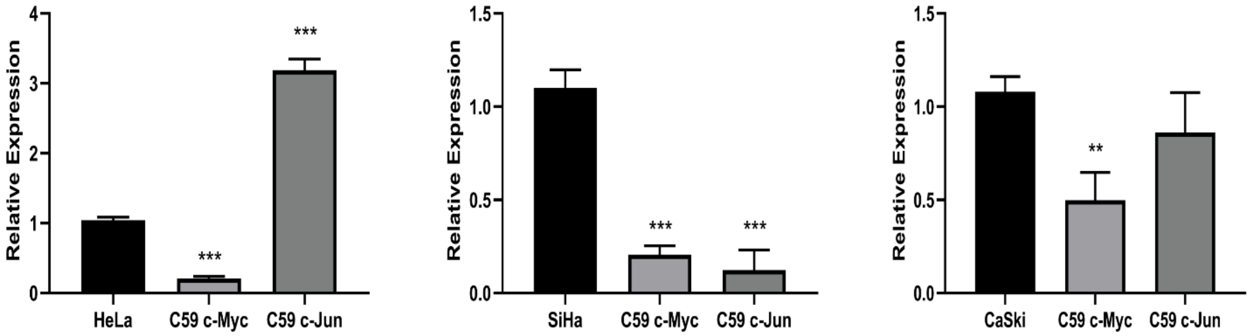

B)

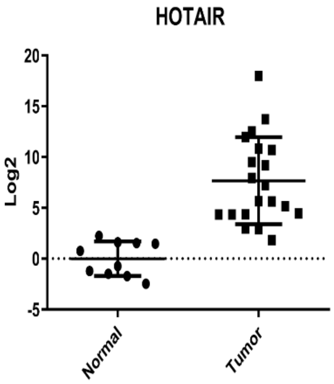

C)

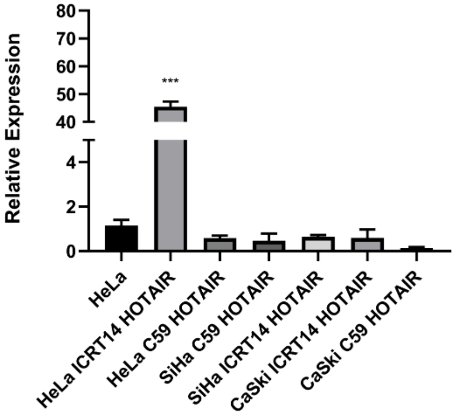

Supplementary File 4. Effect of NSC668036 on Wnt/ß-catenin pathway in CC cell lines.

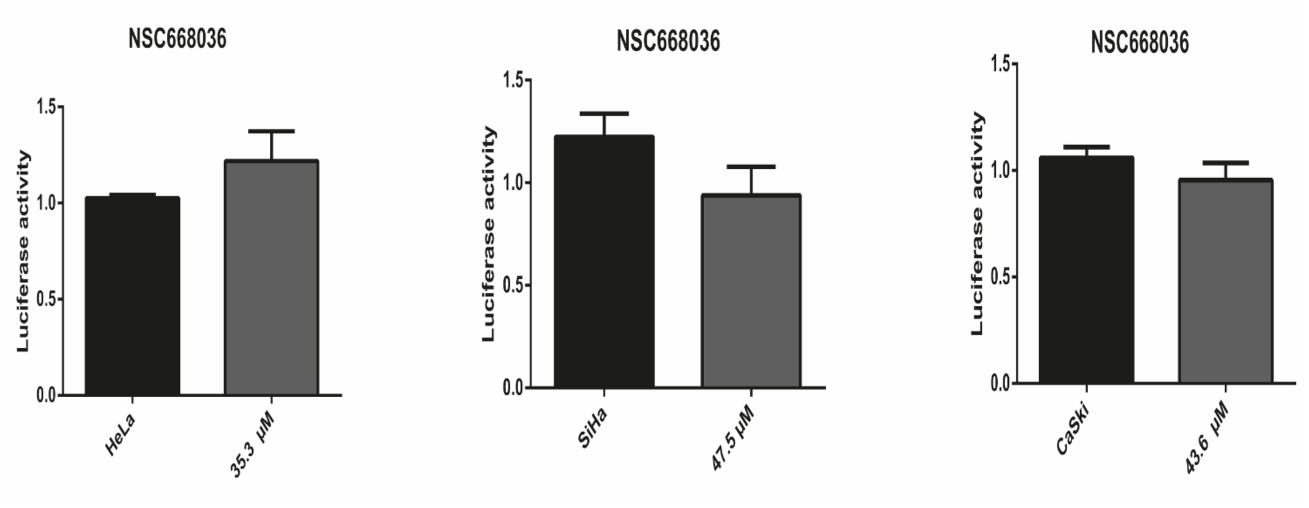

Supplementary File 5. Protein levels of c-Myc and c-Jun detected by western blot, in HeLa (A), SiHa (B) and CaSki (C) treated with ICRT14, C59 and DsiHOTAIR. ß-actin was used as loading control. (D) Protein levels of PARP1 and Caspase 3 detected by western blot in HeLa cells treated with ICRT14, DsiHOTAIR and DsiHOTAIR plus ICRT14 combination. ß-actin was used as loading control.

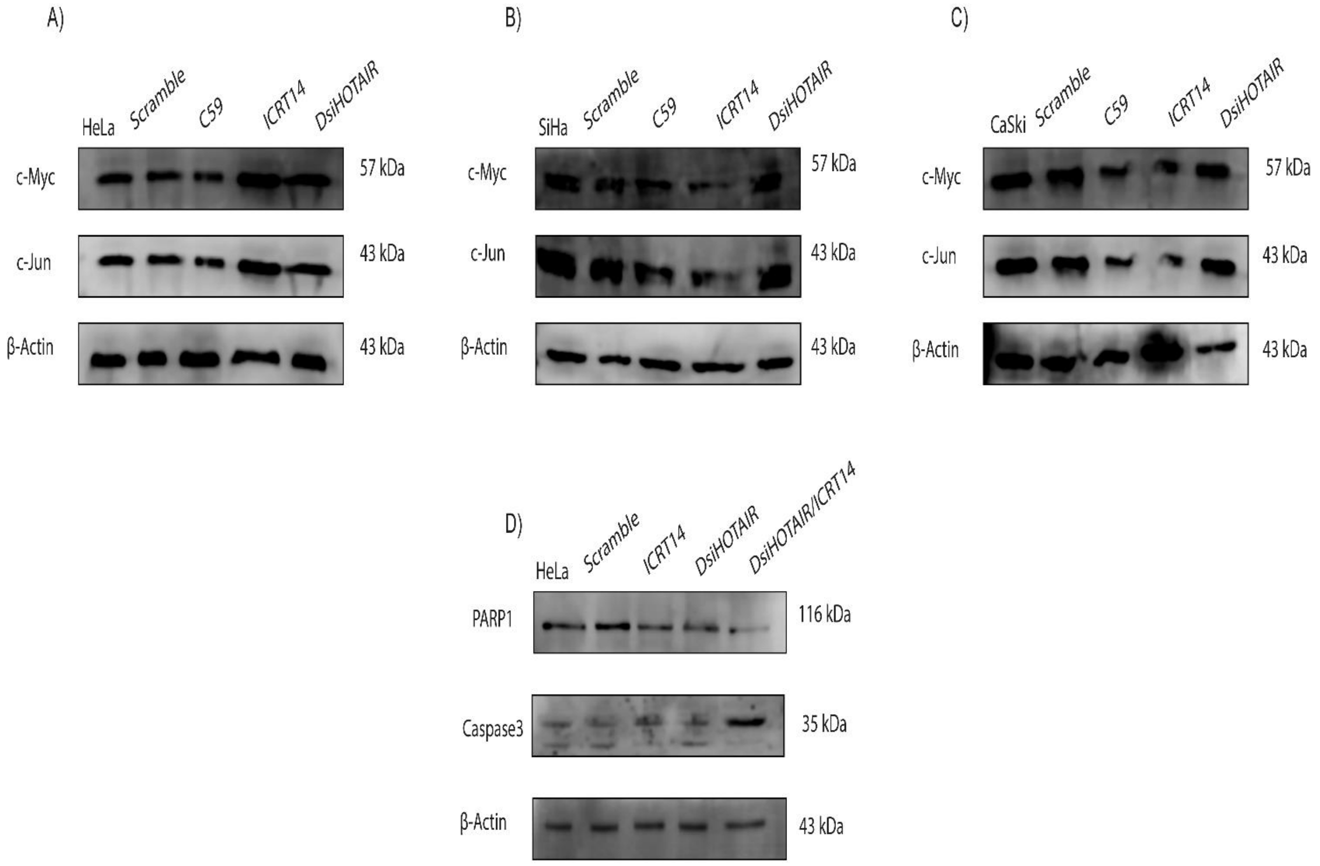

Supplementary File 6. Western blot to IP ß-catenin confirmation of RIP assays. Anti-IgG served as negative control.

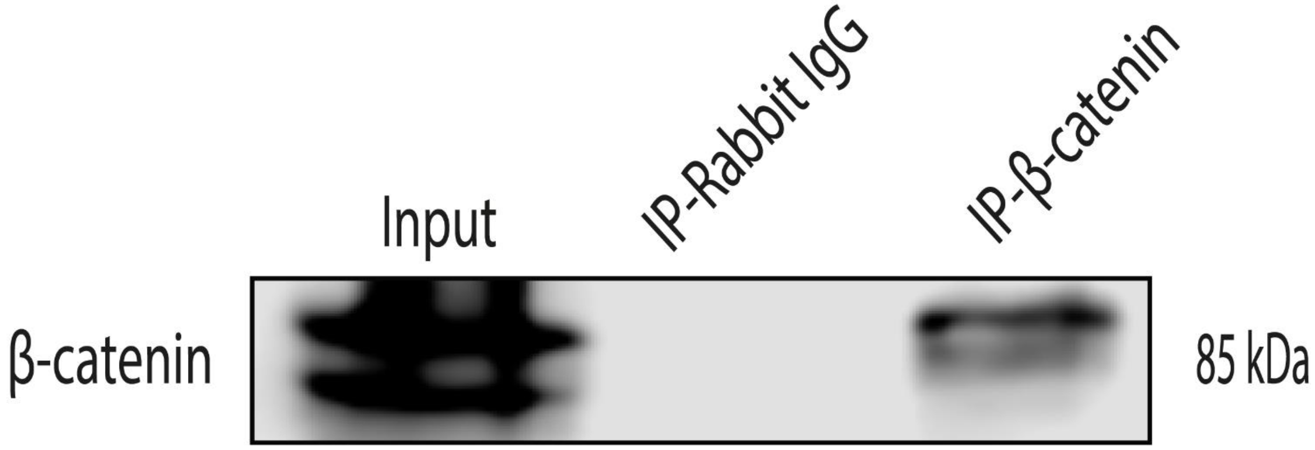

Supplement: Supplementary File 1 — Primer sequences. [file DataSheet_1.pdf]
